# Incidence of major complications related to endotracheal intubation in ICUs of nonacademic Brazilian public hospitals

**DOI:** 10.1186/cc12096

**Published:** 2013-03-19

**Authors:** AB Cavalcanti, K Normilio-da-Silva, JC Acarine Mouro, F Moreira, AA Kodama, R Del-Manto, O Berwanger

**Affiliations:** 1 Hospital do Coração - HCor, São Paulo, Brazil; 2 Hospital Militar de Área de São Paulo - HMASP, São Paulo, Brazil

## Introduction

Our objective is to determine the incidence of major complications related to endotracheal intubations (death, cardiac arrest, severe hemodynamic instability or severe hypoxemia) performed in the ICUs of nonacademic Brazilian public hospitals.

## Methods

This is a report of the baseline phase of a cluster randomized trial with two parallel arms. In this baseline phase, we collected data from sequential patients needing endotracheal intubation in 17 ICUs from Brazil. Patients needing endotracheal intubation after cardiac arrest were excluded. In a second ongoing phase, not reported here, we have randomized ICUs to a multifaceted intervention to prevent complications of endotracheal intubation. Primary outcome was defined as the occurrence of death, cardiac arrest, severe cardiovascular instability (systolic blood pressure <60 mmHg at least one time, <90 mmHg and persisting >30 minutes or need to initiate vasopressors) or severe hypoxemia (SpO_2 _<80%) within 1 hour after intubation.

## Results

We evaluated 246 intubations. The mean age was 49 ± 21.9 years; the SAPS 3 admission score was 57.4 ± 15.4. The most common indications of intubation were acute respiratory failure (61.0%) and coma (17.1%). The primary outcome occurred in 138 of 246 intubations (56.1%). Within the first hour, the incidence of severe hypotension was 47.2%, of severe hypoxemia was 20.3%, of cardiac arrest was 5.7% and of death was 4.5%.

## Conclusion

Incidence of major complications associated with endotracheal intubation is unacceptably high in Brazilian public ICUs. There is an urgent need for effective measures to increase safety of this common procedure in our environment.

